# Advances in the treatment of autosomal recessive congenital ichthyosis, a look towards the repositioning of drugs

**DOI:** 10.3389/fphar.2023.1274248

**Published:** 2023-11-09

**Authors:** Sheila I. Peña-Corona, Stephany Celeste Gutiérrez-Ruiz, Ma de los Dolores Campos Echeverria, Hernán Cortés, Manuel González-Del Carmen, Gerardo Leyva-Gómez

**Affiliations:** ^1^ Departamento de Farmacia, Facultad de Química, Universidad Nacional Autónoma de México, Ciudad de México, Mexico; ^2^ Laboratorio de Medicina Genómica, Departamento de Genómica, Instituto Nacional de Rehabilitación Luis Guillermo Ibarra Ibarra, Ciudad de Mexico, Mexico; ^3^ Facultad de Medicina, Universidad Veracruzana, Ciudad Mendoza, Veracruz, Mexico

**Keywords:** autosomal recessive congenital ichthyosis, ichthyosis, drug repositioning, gene therapy, skin

## Abstract

Autosomal recessive congenital ichthyoses (ARCI) are a skin pathology due to genetic causes characterized by a variable degree of desquamation, accompanied by erythema. The degree of symptoms is variable, different altered genes are involved, and the symptoms drastically affect patients’ quality of life. Topical treatments are a first-choice strategy due to their ease of application and cost; however, enteral administration of retinoids offers greater efficacy, although with certain limitations. Despite the treatment alternatives, ARCI will persist throughout life, disabling people. Therefore, the search for new treatments always remains necessary. Especially repositioning drugs could be a short-term alternative to new affordable treatments for patients. Taking advantage of extensive knowledge of known drugs or biologics could ensure more accessible and possibly lower-cost treatments. This review briefly and concisely addresses possible repositioning strategies with drugs and biologics for ichthyosis.

## 1 Introduction

Hereditary ichthyoses follow Mendelian inheritance patterns caused by epidermal component gene mutations. Moreover, although different genotypes cause a relatively limited spectrum of clinical phenotypes, they manifest as multiple phenotypic features (skin cornification disorders related to defects in keratinocyte differentiation and epidermal barrier dysfunction) (DiGiovanna and Robinson-Bostom, 2003; Schmuth et al., 2013; Gutiérrez-Cerrajero et al., 2023). Non-syndromic forms of ichthyosis involve autosomal recessive congenital ichthyoses (ARCI), which include lamellar ichthyosis (LI), congenital ichthyosiform erythroderma (Pigg et al., 1998), harlequin ichthyosis (HI), self-healing collodion (SCCB), acral SCCB and swimsuit ichthyosis (Rodríguez-Pazos et al., 2013).

ARCI belongs to the well-known group of rare and orphan diseases and represents a clinical and regulatory challenge for the healthcare sector. Significant barriers, such as low prevalence and severity of the disease, small and heterogeneous patient populations, difficulties in patient recruitment, and limited knowledge of the natural history of the disease, limit the development of new drugs for this population (Fonseca et al., 2019). One strategy proposed to address this challenge is repurposing drugs, a cost-effective and time-saving method compared to developing new orphan drugs, resulting in higher success rates (Roessler et al., 2021).

This review aims to collect information about advances in the treatment of ARCI, which drugs have been proposed as candidates to be reused as new targeted treatments for ARCI. The analysis is based on the inflammatory process recently described in the clarification of the immunological profile reported in patients, the description of the pathophysiological process, prevalence patterns, the conventional treatments currently used in clinical practice, as well as advances in corrective gene therapy.

## 2 Epidemiology of ARCI

Although mutations in the transglutaminase 1 (TGM1) gene have been identified worldwide (Herman et al., 2009), their prevalence is relatively low, at 1:100,000 population (Vahlquist et al., 2008). However, the figures may vary from one geographical region to another. In this regard, an epidemiologic study in France reported a prevalence of ARCI of 7:1,000,000 (Dreyfus et al., 2014). In contrast, in other areas, such as Norway, the prevalence is 1:91,000 (Pigg et al., 1998), and on the Galicia coast, it is about 1:33,000 (Rodríguez-Pazos et al., 2011), thus, the population affected is higher than other rare diseases such as genodermatosis. In North Cairo, a clinical trial indicated a 1:2,359 rate for genodermatosis in patients at the Children’s Hospital, Ain Shams University, Cairo, Egypt. The high prevalence correlated with the consanguineous marriages reported when interviewed and registered in previous studies (El-Sayed et al., 2018).

The overall prevalence of ARCI in the United States has been reported to be 1:200,000–300,000 (Bale and Doyle, 1994). However, in some parts of Spanish-colonized Latin America, the frequency of ARCI is high. The northwestern coast of Ecuador has a prevalence of 1:50,000 (Esperón-Moldes et al., 2019); and recently, Gonzalez et al. described a significant presence of settlers with the disease in Veracruz State, Mexico, mainly in the community of El Campanario where the prevalence is about 2,352:100,000 (González-Del Carmen et al., 2020), probably the highest reported worldwide. The existence of a founder effect and traditional parental consanguinity in each area could explain the discrepancy in figures.

## 3 Physiopathology of ARCI

ARCI has a broad clinical spectrum with variable severity (Vahlquist et al., 2018). The alteration of the skin barrier is mainly manifested by thick, dark brown, generalized scales accentuated on the lower extremities and flexures, accompanied by scaling and erythema in severe cases, resulting in increased transepidermal water loss (Ali et al., 2013; Vahlquist et al., 2018; Gutiérrez-Cerrajero et al., 2023). Oral involvement (Ramar et al., 2014; Nair and Kodhandram, 2016), hypoacusis (Huang et al., 2014), palmoplantar hyperlinearity, diffuse yellowish palmoplantar keratoderma, ectropion, pruritus, alopecia, digital contractures, subungual hyperkeratosis, onychogryphosis, keratosis pilaris, malformation of the nasal cartilage, severe inflammation with a specific immunologic profile and frequent births as collodion babies have also been described (González-Del Carmen et al., 2020; Vahlquist et al., 2018; Süßmuth et al., 2020; Paller, 2019). Stunting is another consequence of increased caloric and nutrient requirements due to epidermal deficiency (Vahlquist et al., 2018).

Several mutations in different genes are responsible for the clinical manifestations (Joosten et al., 2022), generally encoding epidermal proteins involved in cornified cell envelope (CCE) formation, cytoskeleton, lipid metabolism, DNA repair, cell–cell junction proteins, and enzymes required for the proteolysis of cell junctions (Süßmuth et al., 2020).

Mutations in TGM1 gen which encodes for TGM1 protein, are the most common cause of ARCI (Pigg et al., 1998). TGM1 enzyme catalyzes the cross-linking of Nε-(γ-glutamyl)lysine from precursor proteins and ω-hydroxyceramides while forming the cytoplasmic layer of the CCE (Herman et al., 2009). However, Elias et al. reported an apparent accumulation of covalently bound lipids even without functional TGM1 activity (Elias et al., 2002). This still represents a gap in understanding the pathophysiological pathways of ARCI. Furthermore, an insufficient correlation between the mutations and the specific phenotype has been reported (Oji et al., 2010; Metze et al., 2021). Therefore, ARCI represent a challenge for medical treatment and the search for pharmacological solutions.

## 4 Conventional treatments for ichthyosis

Usually, conventional treatments for ichthyosis are the first approach for symptomatic relief because they are more readily available, less expensive, and have relatively well-known side effects. Conventional treatments range from physical removal of scales to systemic administration of pharmacological products to achieve an easy, better, and more lasting symptomatology control of the illness (Springer, 2004) (Table 1).

**TABLE 1:** Common agents used in conventional therapy for ichthyosis with some side effects referred.

Therapy	Indications/active substance	Frequency/Dosage	Limits/Side effects	References
Bathing	Daily bath	Up to twice daily 10 min	Depending on symptom severity and availability	Shwayder (2004), Traupe and Burgdorf (2007), Oji and Traupe (2009)
	Bath with Soaking Soak for 15–30 min	Up to twice daily At least once a week		DiGiovanna and Robinson-Bostom (2003), Shwayder, 2004; Springer (2004)
	Bath deep with Soaking 15–30 min plus an additive (salt, sodium bicarbonate, wheat, corn, or rice starch to bath)	3–7 times per week	Depending on symptom severity and availability Salt could be irritating	DiGiovanna and Robinson-Bostom (2003), Traupe and Burgdorf (2007), Oji and Traupe (2009)
	Steam bath, 15–30 min	Twice a week or daily	Depending on symptom severity and availability	Traupe and Burgdorf (2007), Oji and Traupe (2009)
Mechanical scale removal	Repetitive gentle rubbing Apply in all baths using microfiber household towels, or pumice stones	During bath/steam bath (Soak for 15–30 min)	It can hurt the skin	Traupe and Burgdorf (2007), Oji and Traupe (2009)
Keratolitics	Urea	≥10%	Avoid use during the first year of life	DiGiovanna and Robinson-Bostom (2003), Cortés et al. (2020)
	Salicylic acid	0.5%–3%	Contraindicated in infants and should only be used on small areas in children or adults, it may produce salicylate toxicity	Traupe and Burgdorf (2007), Vahlquist et al. (2008), Cortés et al. (2020), Süßmuth et al. (2020)
		5%	Whole-body treatment and use in children are contraindicated	
α- hidroxy acids	Lactic acid	≤12% topical formulation applied twice daily	Aged ≥2 years Adverse events: Erythema, irritation, stinging/burning Limit use on areas that may be exposed to ultraviolet (UV) light	Springer (2004)
	Glycolic acid	5%–12%		Cortés et al. (2020)
Others	Propylene glycol	>20% 30%–50% (in water)	Used in specific areas, with special instructions and periods of time	DiGiovanna and Robinson-Bostom (2003), Shwayder (2004)
	N-acetylcysteine	10% combined with urea 5%	Irritation, odor of sulfur, intolerance	Kaplan and Castelo-Soccio (2018), Cortés et al. (2020)
Emollients	Urea	<5% emollient	Avoid use during the first year of life	DiGiovanna and Robinson-Bostom (2003), Cortés et al. (2020)
	Petrolatum	-	Intolerance	Springer (2004), Vahlquist et al. (2008)
	Propylene glycol	20%–30%	Age >2 years Changes in blood osmolarity are being discussed	Süßmuth et al. (2020), Jaffar et al. (2023)
	Glycerol	5%–10%	Intolerance	Süßmuth et al. (2020)
	Dexpanthenol	5%	Intolerance	Cortés et al. (2020), Süßmuth et al. (2020)
Topical Retinoids	Tretinoin	0.1%	Burning, stinging, peeling, redness, and dryness of the skin. Teratogenicity	Traupe and Burgdorf (2007)
	Tazarotene	0.05%–0.1% Limit use to <10–20% of body area	Local irritation, burning, erythema, and contact dermatitis. Avoid exposure to UV light and pregnancy. Teratogenicity	DiGiovanna and Robinson-Bostom (2003), Springer (2004)
	Adapalene	0.1%	Erythema, local dryness	Mo et al. (2022)
Systemic treatments, oral Retinoids	Acitretin	0.5 mg/kg	Diffuse skeletal hyperostosis, teratogenicity. Acute and chronic toxicities. Xerosis, dry nose, and eye irritation are common. Abnormalities in blood cell counts, chemistries, liver enzymes and lipids	DiGiovanna and Robinson-Bostom (2003), Springer (2004), Traupe and Burgdorf (2007), Vahlquist et al. (2008), Cortés et al. (2020)
	Isotretinoin	1 mg/kg		DiGiovanna and Robinson-Bostom (2003), Springer (2004), Vahlquist et al. (2008)

Topical therapy is one type of conventional treatment and is based on hydration, lubrication, and keratolysis. Its goals are to restore and maintain the skin barrier, diminish transepidermal water loss, and reduce the build-up of scaly skin to alleviate associated symptoms like pruritus by promoting the desquamation of the stratus corneous and enhancing skin hydration (DiGiovanna and Robinson-Bostom, 2003).

The plainest treatment using topical therapy goes from a relatively simple period of soaking (15–30 min) in a water bath, followed by a repeated gentle scrub of the surface of the skin to remove scales. Also, application of topical creams, ointments, gels, or lotions to keep adsorbed moisture up, to hydrate the skin, to lubricate it or to promote its desquamation and avoid infections (Table 1) (DiGiovanna and Robinson-Bostom, 2003; Springer, 2004; Traupe and Burgdorf, 2007).

Due to the variability of types and severity of the disease, some specific emollient and keratolytic agents are used to formulate the products needed to obtain the benefits of each stage of the treatment. The emollient and keratolytic agents can be used as monotherapy, combined in the same or different formulations, or with topical or systemic retinoid therapy (Table 1) (DiGiovanna and Robinson-Bostom, 2003; Springer, 2004; Traupe and Burgdorf, 2007; Cortés et al., 2020).

Even though topical retinoids put the symptoms in remission in a faster and more complete way than the firsthand formulations, the amount of retinoids absorbed through the impaired barrier of ichthyotic skin is larger than in skin with normal conditions causing a higher probability for side effects and risk of teratogenicity given the large area of skin exposed to formulations. Limited percentages of treated skin areas are set, and doses are kept at a minimum to minimize this risk (DiGiovanna and Robinson-Bostom, 2003; Cortés et al., 2020).

**FIGURE 1:**
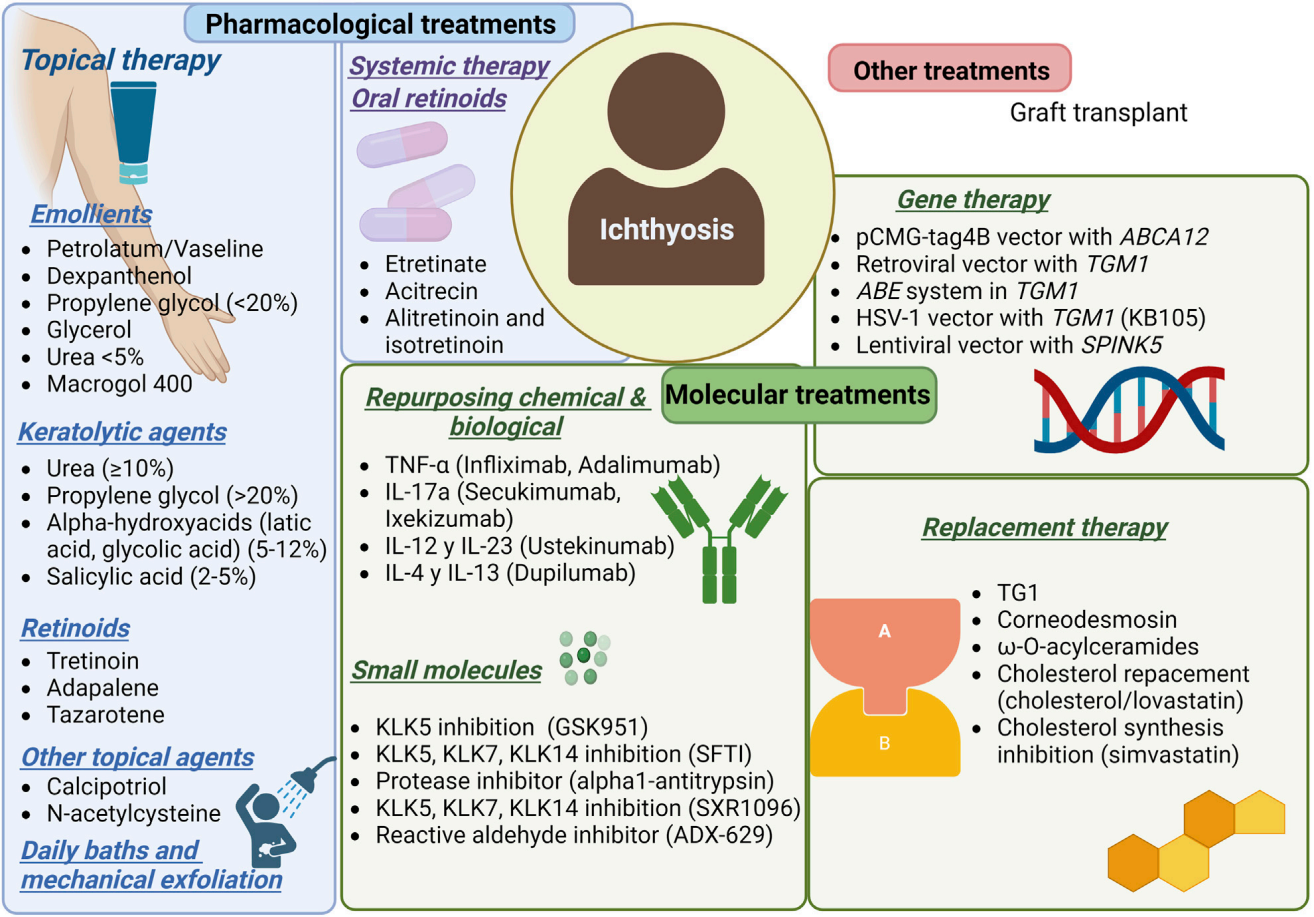
Current treatments for ichthyosis patients.

Almost all patients with severe disease get phenotypic improvement using systemic retinoids within weeks. Retinoids cause a generalized keratolytic effect, they may increase the ability to sweat, ameliorate ectropion, and have anti-inflammatory activity, but the side effects for long-term use can be severe (DiGiovanna and Robinson-Bostom, 2003; Oji and Traupe, 2009; Aufenvenne et al., 2013; Digiovanna et al., 2013) (Figure 1).

The effectiveness of conventional treatments relies on the type and severity of the disease, individual and environmental factors, severity of the symptoms, treatment adherence, time spent for grooming, psychosocial status, and considering that it would be a long-life treatment, economic and social factors matter (Vahlquist et al., 2008).

## 5 Molecular treatments for ichthyosis: Replacement therapy

As mentioned, the major underlying genetic defect is in TGM1. Therefore, it is the first choice for replacement therapy, and nanotechnology has been used to deliver the enzyme TGM1. Aufenvenne et al. developed a liposomal system with encapsulated recombinant human TGM1 (Aufenvenne et al., 2013). The study demonstrated *in situ* restoration of TGM1 activity and normalization of the distribution patterns of many epidermal differentiation markers. The results suggest optimization of the formulation, as the higher dose resulted in epidermal hyperproliferation and TGM1 hyperactivity.

The development of a TGM1-loaded thermoresponsive nanogel achieved protein integrity and bioactivity and restored skin barrier function after application in TGM1-deficient skin models. However, the authors did not report enzyme loading capacity (Witting et al., 2015). Another study published in 2018 confirmed the improved restoration of barrier function in ARCI skin equivalents treated with nanogel (Plank et al., 2019).

On the other hand, kallikreins (Nauroy and Nyström, 2020) may be considered a future replacement therapy [14]. Under homeostatic conditions, these enzymes promote corneocyte desquamation and are part of the regulation of skin innate immunity. However, in certain cutaneous pathological conditions, dysregulation of KLKs leads to skin barrier damage and expression of proinflammatory cytokines (Nauroy and Nyström, 2020). ABCA12 gene mutations result in decreased expression of KLK5 and KLK7 proteases, leading to severe hyperkeratinization, a defect described in the major ARCI of HI (Scott et al., 2013; Zhang et al., 2016). Based on this, Zhan et al. (Zhang et al., 2016) administered a cream containing recombinant KLK5 and KLK7 in a whole skin graft system of HI and showed an evident detachment of the hyperkeratotic SC after 1 week.

Although protein replacement is a novel strategy to address skin barrier dysfunction, it is important to consider that ichthyosis involves multiple enzyme dysregulation and high development costs. Alternatively, another interesting replacement therapy proposed uses epidermal ceramides, specifically ω-O-acylceramides, to restore CCE against structural skin defects associated with PNPLA1 mutation (Borodzicz et al., 2016; Grond et al., 2017). This is a promising and cost-effective approach to treatment.

## 6 Gene therapy for ichthyosis

Ideally, gene therapy would correct the genetic defect that causes ARCI. Unfortunately, very few trials have taken place. Although there have been positive results in preclinical studies, there are still concerns about the safety of gene therapy (Abdul et al., 2014).

The most recent study provides strong support for the safe and effective treatment of human TGM1 via herpes simplex virus type 1, and its successful results are due to the intrinsic properties of the chosen vector (Freedman et al., 2021). A Phase I/II clinical trial (NCT04047732) is ongoing, and this interventional study will evaluate the safety and efficacy in a maximum of 6 subjects for approximately 3.5 months. The study will use the Investigator’s Global Assessment (IGA) and the Visual Index for Ichthyosis Severity (VIIS) scales and imaging. The study will include intra-patient comparisons and placebo-treated target areas.

Another interesting proposal is the transfer of the ABCA12 corrector gene. Akiyama et al. presented positive results in restoring lipid secretion for keratinocyte sheet granules using corrective gene transfer of ABCA12 in cultured keratinocytes from patients with harlequin ichthyosis (Akiyama et al., 2005).

Gene therapy is a promising strategy for treating inherited disorders, but much research remains necessary before it can be considered an actual and viable treatment of ARCI. As a result, there may be delays in the approval of clinical trials by regulatory authorities.

## 7 Repurposing chemical and biological for ichthyosis

Drug repositioning (drug reuse, drug rediscovery, or drug reprofiling) identifies new uses for drugs that have already been approved and are under investigation, implies a lower risk of therapeutic failure and a lower investment of time and capital, making it an advantage in searching for new treatments for conditions that need to be resolved promptly (Pushpakom et al., 2019; Sotiropoulou et al., 2021). Currently, the treatment for ichthyosis focuses on emollients, keratolytic, and oral retinoids. In the last decade, the repurposing of chemical and biological compounds for ichthyosis has been proposed based on Paller et al. studies (Paller et al., 2017; Czarnowicki et al., 2018; Malik et al., 2019; Joosten et al., 2022).

In 2017 Paller et al. (Paller et al., 2017) conducted a study that associate a shared Th (T helper cell) 17/IL (interleukin) −23 immune fingerprint with the major orphan forms of ichthyosis and raise the possibility of IL-17-targeting strategies. The study compared general profile inflammatory markers by analyzing biopsy specimens from 21 genotyped patients with ichthyosis (CIE, LI, epidermolytic ichthyosis, and Netherton syndrome), atopic dermatitis, and psoriasis patients. Samples from ichthyosis patients displayed an increase of general inflammatory (IL-2), innate (IL-1β), and some Th 1/interferon gamma (IFN-γ) markers that were comparable with psoriasis or atopic dermatitis (Paller et al., 2017).

Malik et al. (Malik et al., 2019) conducted a genomic and cellular profiling study to find a fingerprint between humans’ most common orphan ichthyoses, psoriasis, and atopic dermatitis patients. The study performed gene, protein, and serum studies on skin and blood samples from 29 patients (between 2.2 and 56.9 years old) presenting Netherton syndrome, LI, CIE, and epidermolytic ichthyosis. The study included healthy subjects (controls). As a result, the authors found that differentially expressed genes were commonly expressed in all types of ichthyoses; among them, many genes were co-regulated by IL-17, as found by Paller et al. (Paller et al., 2017). Patients with Netherton syndrome exhibited increased T-cell activation compared to the other types of ichthyosis. Transepidermal water loss correlated significantly with regulating IL-17 (IL-17F and IL-36A/IL-36B/IL-36G) gene expression. Thus, this study supports examining IL-17/IL-36-targeted therapies (Malik et al., 2019).

Czarnowicki et al. conducted a study on 47 patients with ichthyosis, among whom 30 were women and 17 were men, between 1 and 57 years of age. 13 of them had LI, 18 CIE, 8 Netherton syndrome, and 8 epidermolytic ichthyoses. Adults in this study had greater T-cell activation than children, which is thought to be due to the chronicity of the disease and the continuous immune stimulation. The authors observed raised IL-17 and IL-22 in all ichthyoses, as in the two previous studies (Paller et al., 2017; Malik et al., 2019). The results indicated activation of IL-17/IL-22 in peripheral blood through ichthyosis. Like Malik et al., T cells characterized ichthyoses, mainly Netherton syndrome and CIE. The authors also observed increased frequencies of T helper 2/cytotoxic T2/Th-9 and similar frequencies of IFN-γ concerning controls (Table 2) (Czarnowicki et al., 2018).

**TABLE 2:** Genetic skin diseases and use of drug repositioning for treatment.

Disease repurposing	Number of participants	Tested compound	Disease original	Treatment	Mechanism of action	References
Ichthyosis	5	Imsidolimab	Generalized Pustular Psoriasis	A starting dose of 400 mg of imsidolimab (day 1), followed by 200 mg every 4 weeks by subcutaneous injection	Inhibits the function of the IL-36-receptor	Trials (2023)
Ichthyosis	13	Ustekinumab	Plaque psoriasis and psoriatic arthritis treatment	Baseline (Day 0) and Months 1, 3, 5, 7, 9, and 11, injections every 8 weeks for 1 year: Month 13, 15, 17, 19, 21, and 23	IL-12 and IL-23 inhibitor	Trials (2021)
Ichthyosis	1	Secukinumab	Moderate to severe plaque psoriasis	300 mg subcutaneously once weekly for 5 weeks, followed by monthly injections	Inhibitors of the Th 17/IL-23 pathway	Haiges et al. (2019)
Ichthyosis	1	Ustekinumab	Plaque psoriasis and psoriatic arthritis treatment	0.75 mg/kg administered at weeks 0, 4 and then 12, every week. Subsequently, the dose was increased to 1.5 mg/kg every 8 weeks	IL-12/IL-23 inhibitor	Poulton et al. (2019)
Alopecia areata	-	Tofacitinib	Psoriatic arthritis	Doses 5 mg and 10 mg	Inhibits JAK3 and JAK1 and, to a lesser extent JAK2	Sotiropoulou et al. (2021)

The above studies demonstrate that in ichthyosis diseases, there is a link between functional barrier inadequacy and inflammation. The mechanisms by which ichthyosis is generated are not yet fully understood. However, specific elevations of Th17/IL-23 pathway cytokines and chemokines have been found in patients’ skin. The strong Th17 bias in ichthyoses is disease-specific and is not a typical phenotype for chronic inflammatory pathologies, even with barrier abnormalities. However, another consideration is that the Th17 bias is an organismal response and compensatory protective attempt leading to inflammation and desquamation (Paller, 2019).

The Th17/IL-17 pathway has a complex autocrine regulation with high plasticity to the environment. Skin barrier defects activate plasmacytoid dendritic cells (pDCs) in the dermis. The pDCs then secrete interferon alpha (IFN-α) and tumor necrosis factor-alpha (TNF-α), which activate classical dendritic cells (cDC). The cDC produces IL-12 and IL-23, specific polarizing cytokines that direct the generation of virgin T cells into Th1 and Th17 cell subsets (Fletcher et al., 2020; Bugaut and Aractingi, 2021). Th17 is differentiated from virgin CD4 T cells by stimulation with IL-6 and transforming growth factor beta (TGF-β); IL-1β and TNFα amplify the process. The differentiation, survival, and proliferation of Th17 cells are dependent on IL-23. IL-23R is induced in Th17 by IL-6 signaling through Janus kinases (JAK), JAK1, JAK2, and tyrosine kinase 2 (TYK2), signal transducers and activators of transcription 3 (STAT3) and RAR-related orphan gamma t (RORγt). IL23 signals through JAK/STAT3, leading to a Th17 phenotype and upregulation of RORγt expression, which promotes IL-17 secretion (Bugaut and Aractingi, 2021).

In addition to Th17 cells, IL-17A and IL-17F are also secreted by CD8/Tc17 T cells, innate lymphoid cells type 3 (ILC3), invariant natural killer (iNK) T cells, gamma-delta (γδ) T cells, mast cells, Paneth cells, and possibly myeloid cells and neutrophils (Fletcher et al., 2020; Bugaut and Aractingi, 2021; Mills, 2023). Many of these cells depend on IL-23 (Fletcher et al., 2020; Ten Bergen et al., 2020; Bugaut and Aractingi, 2021). IL-23 is a heterodimer of p19 and p40, with the p40 subunit shared with IL-12, a major Th1 cell driver (Fletcher et al., 2020).

Th17 cells produce not only IL-17A and IL-17F but also IL-22, which alone or together with TNF-α strongly activate keratinocytes and act synergistically to induce a proinflammatory signaling cascade (Bartlett and Million, 2015; Fletcher et al., 2020). Homodimers or heterodimers of IL-17A and IL-17F bind to a receptor composed of RA and RC subunits but with different affinities. Subsequently, keratinocytes secrete antimicrobial peptides, cytokines, and chemokines that recruit and activate immune cells, resulting in a feedback loop of persistent inflammation, epithelial cell proliferation, and hyperkeratosis (Fletcher et al., 2020; Bugaut and Aractingi, 2021). In addition, IL-17A induces the production of the chemoattractants CXCL1, CXCL2, and CXCL8, which attract neutrophils and macrophages, and chemokine ligand 20 (CCL20), which captures Th17 and conventional DC (cDC). The same synergistic axis positively regulates the expression of IL-36, which then increases the function of Th17 cytokines and stimulates keratinocyte proliferation (Ten Bergen et al., 2020).

**FIGURE 2:**
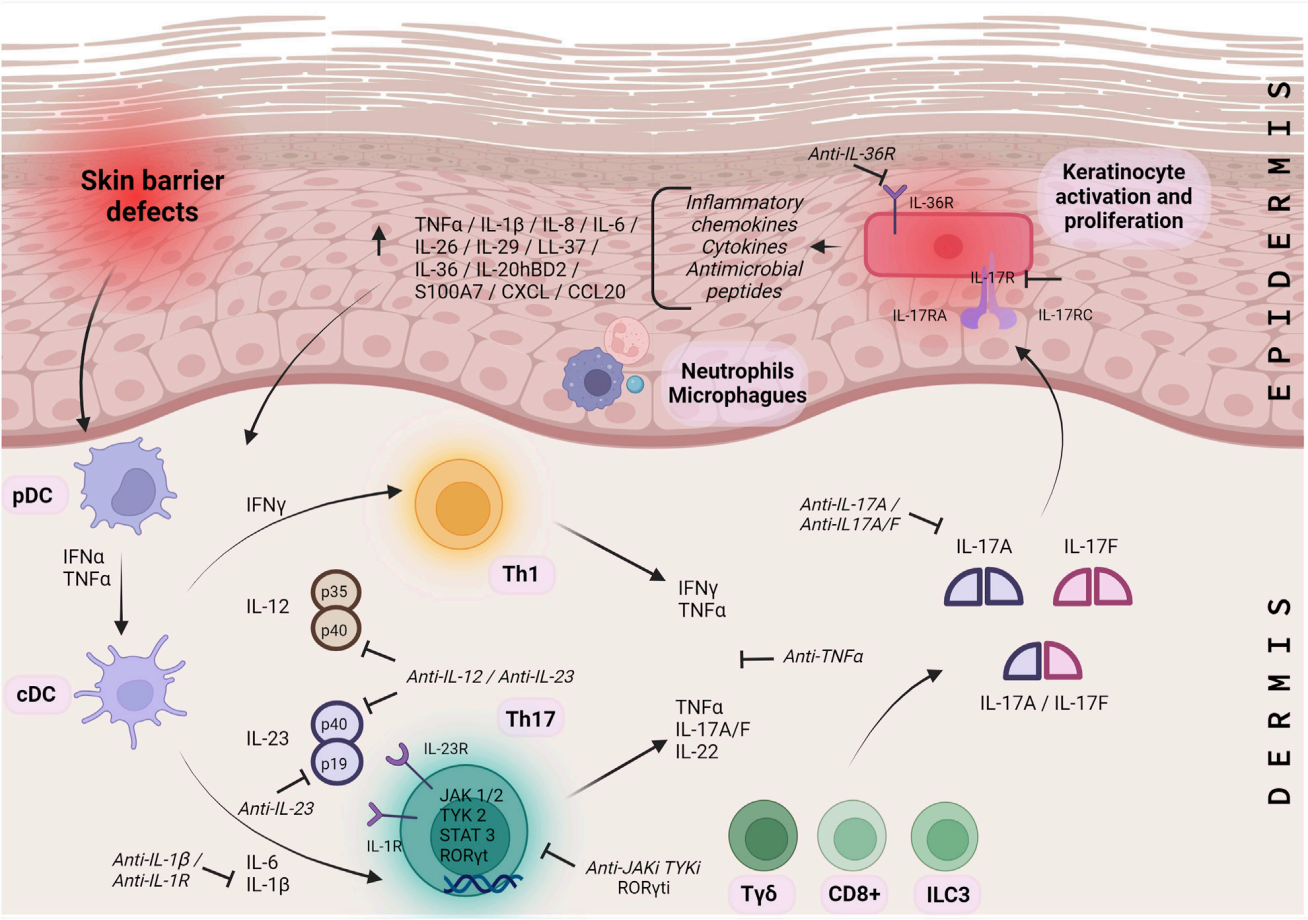
Molecular pathways of ARCI inflammation. Molecular pathway IL-23/JAK/STAT3/RORγt/IL-17 representing the inflammatory process for ARCI’s. pDC: plasmacytoid dendritic cells; cDC: dendritic cells; Th: T helper cells; Tγδ: gamma-delta T cells; CD+: cluster of differentiation positive T cells; ILC: innate lymphoid cells; HS: hidradenitis suppurativa; IL: interleukin; IFN: interferon; TNF: tumor necrosis factor alpha; TGF: transforming growth factor beta; JAK: janus kinases; TYK 2: tyrosine kinase 2; STAT3: signal transducers and activators of transcription; RORγt: RAR: related orphan gamma t; IL-R: interleukin receptor.├: Proposed therapeutic targets.

The complete IL-23/JAK/STAT3/RORγt/IL-17 pathway plays a critical role in understanding the inflammatory process of ARCI and is an important target for many recent or emerging therapies for ichthyosis (Figure 2).

Currently, the repurposing drugs in ichthyosis are focused on the use of drugs that inhibit factors related to immune system molecules such as TNF-α (infliximab, adalimumab), IL-17A (Secukinumab, Ixekizumab), IL-4, IL-12, IL-23 (Dupilumab), and IL-36 (Paller, 2020; Trials, 2021; Joosten et al., 2022; Trials, 2023).

Ongoing clinical trials are studying ichthyosis in humans using drug repositioning. One of them, currently involving 13 individuals, conducted by Dr. Paller at Northwestern University, is studying treatment with ustekinumab, where subjects receive injections every 8 weeks. The treatment will be carried out for 1 year. The aim is to find out if this drug, which is superimposed, has a reduction in the severity of the condition and to analyze how safe this drug is for patients during its use and the occurrence of bacterial and fungal infections (Trials, 2021).

In another clinical trial, Imsidolimab was also studied as a repositioning drug in ichthyosis, culminating on 19 November 2021, in the United States. The immune response was explored in patients who received a 400 mg dose of imsidolimab from day 1, and subsequently, 200 mg of imsidolimab was administered every 4 weeks by subcutaneous injections. The objectives were to characterize the pharmacokinetic profile of imsidolimab and investigate the immune response to this drug in ichthyosis participants. The study was ended because of insufficient recruitment of participants. However, there are reports of adverse events such as fatigue, herpes simplex reactivation, and headache (Trials, 2023).

**FIGURE 3:**
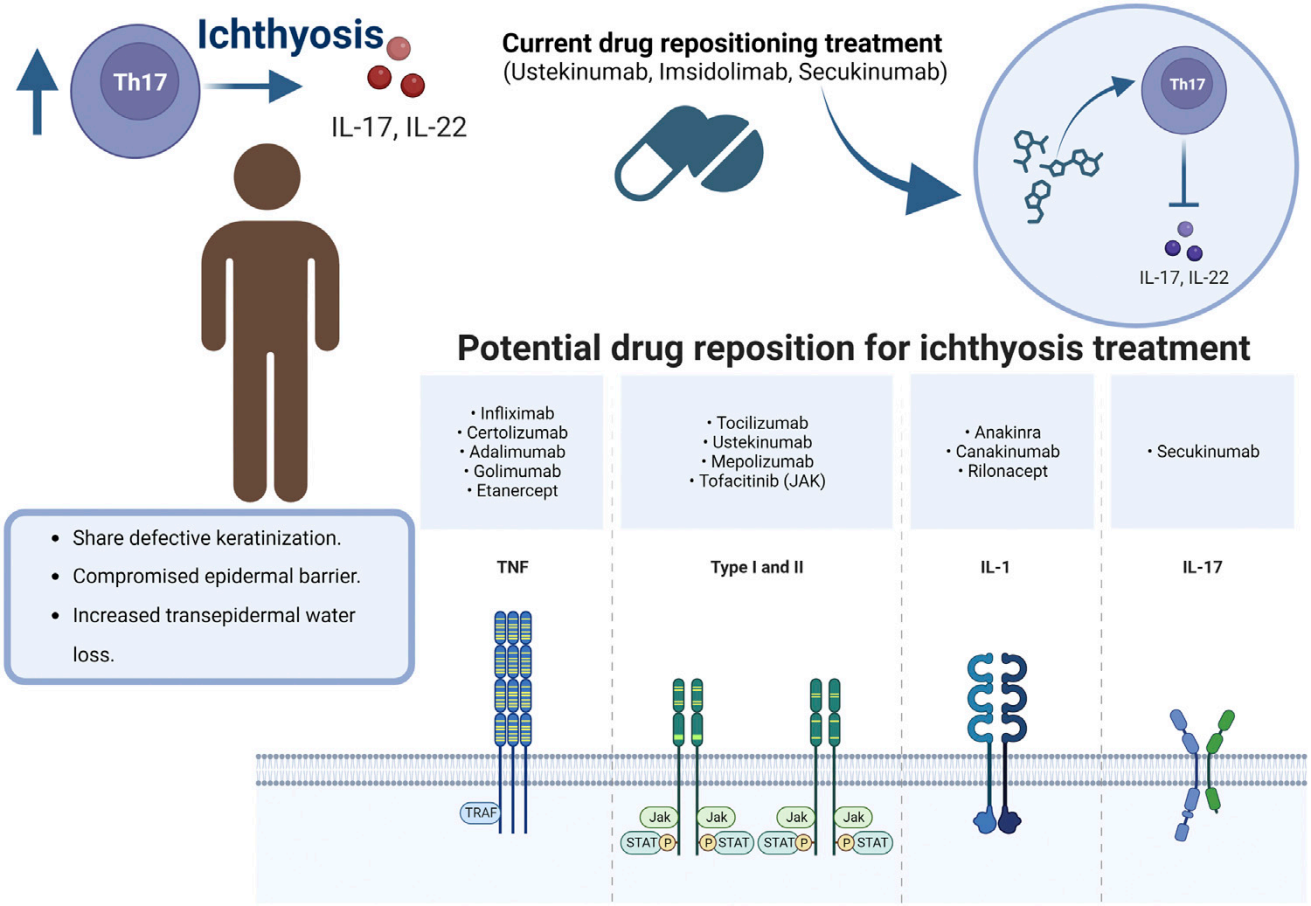
Drug repositioning for Ichthyosis.

Secukinumab (Th17/IL-23 inhibitor) was administered to a 20-year-old man with ARCI-CIE, arthritis, and chronic liver disease. Scaling and erythroderma were reduced 2 months later, but acanthosis, hyper-, and parakeratosis were slightly reduced. No secondary effects associated with anti-IL-17 therapy were reported (Haiges et al., 2019). Ustekinumab (IL-12/IL-23 inhibitor) was evaluated in a 4-year-old boy with ARCI (Poulton et al., 2019) to mitigate the clinical expression of ichthyotic disease. There was reported that the patient had a favorable dramatic response to ustekinumab, and an improvement in his quality of life when the ustekinumab was administered every 8 weeks, as opposed to the period of every 12 weeks used in psoriatic patients (Figure 3). Thus, the dose and period of administration frequency are challenging in antibodies directed to ichthyosis disease.

There are limited studies that evaluated drug repositioning in ichthyosis. However, in other skin inflammatory disorders, dupilumab and secukinumab have been applied in patients with atopic dermatitis (Cada et al., 2015; Paller, 2020). In another example, research has been conducted to introduce repurposed drugs to treat genodermatoses, an inherited skin disease. Correcting the consequences or genetic defect may require personalized strategies depending on the nature of the genodermatoses, the protein involved, or even the mutation. The reused drugs, rather than a therapy, can be a strategy, especially when the defect is at the genetic level (Morren et al., 2021). Other examples of repurposing drugs directed to immune molecules are tofacitinib and ruxolitinib, a JAK used in alopecia. In alopecia areata in mice and humans, gene expression signatures indicative of IFN- γ responses and cytotoxic T-cell infiltration have been found. Since Janus kinases are upstream effectors of IFN-γ and γc cytokine receptors, it is hypothesized that JAK inhibitors could induce hair growth (Sotiropoulou et al., 2021).

Drug repurposing for ichthyoses must be directed to intervene where cell-signaling pathways are dysregulated for direct or indirect modulation of signaling pathways and recover the adequate functioning of skin cells. Although dysregulated immune molecules in ichthyosis disease are currently reported, there are lack of clinical studies in ichthyosis patients to determine the doses and the period of administration of repurposing drugs.

## 8 Conclusions and perspectives

The central axis of repositioning for ARCI points to the modulation of inflammatory processes with the regulation of the participation of TNF-a, IL-4, IL-12, IL-17a, IL-23, and IL-36. Especially the use of ustekinumab, imsidolimab, secukinumab is in phases of clinical trials with ichthyosis patients. It highlights that, as in all pharmacological administration, the dose and frequency are the main challenges to establish while maintaining patient safety. Although the outlook for repositioning drugs for ichthyosis is still limited, it is promising because it is a low-frequency pathology. All interventions to modulate inflammatory processes for ichthyosis must be controlled and directed to preserve immune integrity and reduce side effects.
